# Evaluation of konjac noodle as a microsurgery training model: learning curve analysis

**DOI:** 10.1590/0100-6991e-20233528-en

**Published:** 2023-06-22

**Authors:** TIAGO MARQUES AVELAR, RENAN MAXIMILIAN LOVATO, THIAGO GOMES BARBOSA, PAULO ADOLFO WESSEL XANDER, LEONARDO HENRIQUE DA SILVA RODRIGUES, ADRIANA JOSE BRITO CAMPOS, RICARDO SALEMI RIECHELMANN, JUAN ANTONIO CASTRO FLORES, GUILHERME BRASILEIRO DE AGUIAR, JEAN GONÇALVES DE OLIVEIRA, JOSÉ CARLOS ESTEVES VEIGA

**Affiliations:** 1 -Faculdade de Ciências Médicas, Santa Casa de São Paulo, Departamento de Cirurgia, Divisão de Neurocirurgia - São Paulo - SP - Brasil

**Keywords:** Learning Curve, Microsurgery, Simulation Training, Anastomosis, Surgical, Curva de Aprendizado, Microcirurgia, Treinamento por Simulação, Anastomose Cirúrgica

## Abstract

**Background:**

classical models of microsurgical anastomosis training are expensive and have ethical implications. Some alternatives join low cost and easiness to store. However, the translation of knowledge acquired by training in these methods into the traditional ones is not clear. This project aims to assess the feasibility of konjac noodles as a reliable microsurgery-training model.

**Methods:**

10 neurosurgery residents performed an end-to-end anastomosis in a 2-3mm placenta artery. The anastomoses were evaluated quantitatively, recording time; and qualitatively, applying a validated score (Anastomosis Lapse Index - ALI) by three experienced neurosurgeons and verifying the presence of gross leakage through the infusion of fluorescein. Subsequently, they performed 10 non-consecutive sessions of anastomosis training in the konjac noodle. Eventually, a final anastomosis in the placenta model was performed and the same parameters were scored.

**Results:**

we observed a 17min reduction in the mean time to perform the anastomosis in the placenta model after the training in the konjac (p<0.05). There was a non-significant 20% reduction in gross leakage, but the training sessions were not able to consistently improve the ALI score.

**Conclusions:**

we demonstrate a reduction in anastomosis performing time in placental arteries after training sessions in the konjac noodle model, which can be regarded as a feasible low-cost method, particularly useful in centers with surgical microscopes only in the operation room.

## Abstract (pt)

**Introdução:**

modelos tradicionais de treinamento de anastomose microcirúrgica costumam ter custos elevados e implicações éticas de aquisição e manutenção, buscando-se alternativas que reúnam baixo custo e facilidade de armazenamento. Existem diferentes propostas, porém há poucas evidências de que conhecimentos adquiridos com o treinamento nessas plataformas se traduza em melhora na performance, quando estes são comparados a modelos consagrados. Este projeto objetiva avaliar a viabilidade do macarrão de konjac como modelo confiável de treinamento microcirúrgico.

**Métodos:**

10 residentes de neurocirurgia realizaram uma anastomose término-terminal em artéria placentária humana de 2-3mm. As anastomoses foram avaliadas quantitativamente, registrando-se o tempo de confecção e qualitativamente, aplicando-se um escore validado (Anastomosis Lapse Index – ALI) por neurocirurgiões experientes e verificando-se a presença de vazamento grosseiro através da visualização no modo fluorescente injetando-se fluoresceína. Subsequentemente, realizaram 10 sessões de treinamento não consecutivos de anastomose términoterminal no modelo de konjac. Por fim, uma anastomose final foi realizada no modelo placentário e os mesmo parâmetros reavaliados.

**Resultados:**

observamos uma redução de 17 min no tempo médio de confecção da anastomose no modelo de placenta após os treinos no modelo do macarrão (p<0.05). Houve uma redução não significativa de 20% no vazamento grosseiro. As sessões de treino no macarrão não foram capazes de melhorar consistentemente o score ALI.

**Conclusão:**

o treinamento em modelo de macarrão konjac é capaz de reduzir o tempo para realização das anastomoses no modelo em placenta humana, mostrando-se alternativa viável de baixo custo e manutenção, útil em serviços que disponham de microscópio apenas no ambiente cirúrgico.

## INTRODUCTION

Still in the 18^th^ century, Lambert, after discussing a case, wrote in a letter: “If it should be found by experience that a large artery when wounded may be healed up by this kind of suture, without become impervious, it would be an important discovery in surgery”^1^. It took decades, however, for this objective to be achieved, with the first record of a venous suture occurring in 1816. Even though it belonged to the theoretical level at the time, the citation highlights an important characteristic of vascular anastomoses - patency -, which only properly trained surgeons can perform successfully. Currently, this technique is used in several specialties, including ophthalmology, plastic, vascular, and hand surgery, just to name a few. In particular, neurosurgery residents need to acquire microsurgical skills.

Some issues, however, have been limiting the access of young surgeons to complex cases. Among them, there are the growing number of patients treated by endovascular approaches and the concentration of challenging vascular cases in very few institutions. Therefore, the development of training models that simulate the real conditions found in the surgical field is fundamental. To achieve this goal, surgeons seek different methodologies, from improving the use of classic models, such as cadaver training^2^, to the use of live biological models (mainly rats)^3^ and, more recently, simulation in virtual reality^4^ ^,^ ^5^. All these examples have advantages and disadvantages. Ethical issues, high cost, and complex logistics can be mentioned as the biggest limitations for the first two. The lack of tactile feedback, essential for fine motor development, and the high level of artificiality are the latter’s biggest drawbacks. Consequently, a model closer to the ideal should combine low cost, ease of storage, absence of ethical conflicts, and the indispensable tactile experience.

In this scenario, a type of noodle used in Japanese cuisine, made from a tuber known as konjac (Amorphophallus konjac)^6^ has been described as a microsurgical training model^7^ ^,^ ^8^, with the advantages mentioned above. However, the theoretical transfer of skills acquired from training in this method in relation to other extensively validated ones has not been previously tested. Thus, this study aimed to evaluate the viability of konjac noodle as a training method in vascular microsurgery, verifying whether the skills acquired in its use as a model would be confirmed in the improvement of anastomosis construction in an already well-established model - the human placenta.

## METHODS

This is an experimental, prospective study carried out in the Neurosurgery laboratory at Santa Casa de São Paulo. Residents in training at the Santa Casa de São Paulo Neurosurgery Service, from the first to the fifth year, were included voluntarily, totaling n=10 individuals. None of the residents had previous training in microsurgical vascular anastomosis.

The participants received an introductory theoretical class on the surgical technique of vascular anastomoses to standardize the basic knowledge in performing microsurgical sutures. Initially, they only trained to make stitches in synthetic material such as latex and silicone^9^, to become familiar with the instruments and the making of microsurgical sutures.

**Figure 1:**
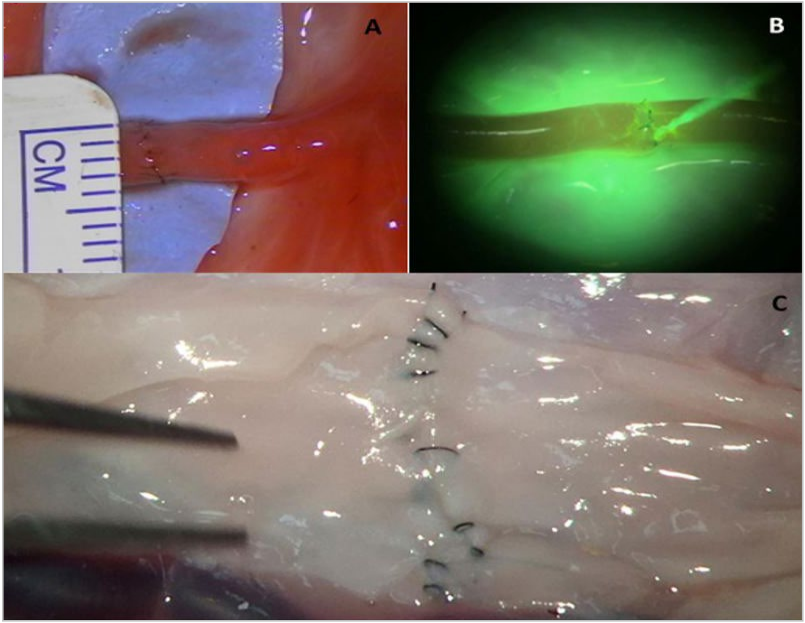
(A) End-to-end anastomosis in placental artery; (B) Under fluorescence mode, after injection of 10mL of fluorescein, gross leakage can be seen through a gap in the anastomosis line; (C) An example of a magnified view (25x) of the luminal face of the suture line for ALI analysis.

From then on, each resident performed a first end-to-end anastomosis in arteries of 2-3mm in human placenta (Figure 1A) - the placentas were gathered from the department of Gynecology and Obstetrics of Santa Casa de São Paulo, after obtaining written consent from parturients under the care of that service. Placentas were prepared as previously described^10^ and kept refrigerated for up to 72 hours, being discarded appropriately. The microsurgical method used simple anastomosis sutures, with Nylon 10-0 with a round needle, 3/8 of a circle, under a microscope (OPMI^®^ Pico, Zeiss). This first anastomosis (A0) was duly documented (through photos) and the time for its preparation was recorded.

For qualitative analysis, two criteria were used. First, two neurosurgeons with clinical experience (at least 10 years) in vascular microsurgery judged the anastomoses through the Anastomosis Lapse Index (ALI)^11^. In summary, this score provides a simple way to assess the level of proficiency in making the anastomosis, classifying the individual as beginner, intermediate, or advanced according to the frequency of occurrence of certain errors. For this purpose, the luminal surface is evaluated using a 25x magnification, visualized after a longitudinal cut of the sutured vessel (Figure 1C).

Second, the permeability of the suture line was evaluated for gross leakage through the anastomosis using fluorescein angiography (Figure 1B). For this, we used 10 mL of a powdered fluorescein-diluted solution, instilled from the catheterization of the artery at the level of the umbilical cord, and visualization of the fluorescent mode through a yellow light filter (Spring Yellow Rosco, Stamford, Connecticut, USA) and ultraviolet lighting (UV flashlight, 2950000 lumens, 980000w, JYX^®^).

**Figure 2:**
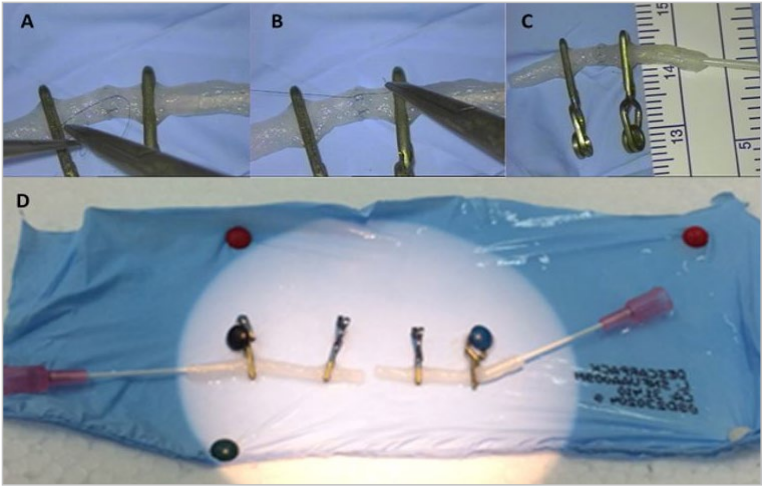
(A,B) Step by step of a stitch being performed on the noodle model. (C) Final appearance of the anastomosis. Note the diameter of the noodle, approximately the same as that of the placental artery. (D) Example of the conceptual base prepared for the training sessions.

After registering this baseline anastomosis, each participant performed 10 non-consecutive sessions (in separate days) of anastomoses in a tubular model prepared in konjac noodles of approximately 3 mm in diameter (Figure 2A-C). To create a tubular model that simulates the wall of a vessel, we modified the technique originally described^7^, piercing the end of the noodle with a peripheral venous catheter with a caliber larger than the one used in the original study (BD INSYTE™ 20G). Two of these segments were positioned facing each other and fixed with aneurysm clips (Vicca™ Neuroclips for laboratory use) and secured through pins on a Styrofoam base, in such a way that the free segments were mobile to allow the anastomosis and, a few centimeters away, its fixed portions simulated the vessels found adhered to the placental surface (Figure 2D). The time of each session was recorded.

After this phase, the residents again performed an anastomosis on the placenta (Af) and the parameters were again recorded (time, ALI, patency). The ALI score was evaluated only after performing the Af, to blind the evaluating neurosurgeons.

To verify the effect of training on a tubular model prepared with konjac noodles on the mean time to perform the anastomosis, we used the paired plot method proposed by Gardner-Altman. In this case, we obtained the paired difference between the means and their respective 95% confidence intervals in both groups after 5,000 bootstraps. To verify if there was a difference in the proportion of procedures with the presence of a visible leakage before and after training, we used the McNemar test. Finally, to assess whether there was any change in the residents’ ALI score before and after training, we used the Cochran test. Regardless of the outcome analyzed, we considered our alpha to be 5%. It is worth mentioning that the methods we selected are used for paired samples, that is, comparison of measurements performed on the same individual at different times. All analyzes were performed using the free Python 3.9 software and using the statsmodels, dabest, pandas, and matplotlib packages.

## RESULTS

**Figure 3:**
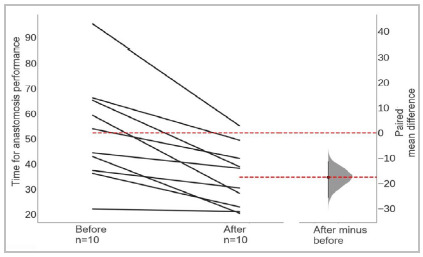
Paired difference in the mean time to perform the anastomosis before and after training (Gardner-Altman plot). Both groups are plotted on the left axis as a slope plot: each observed difference pair is connected by a line. The observed mean difference is plotted on the right axis with its respective distribution and confidence interval after bootstrap.

Regarding the time to perform A0, we observed that, before training, residents performed this procedure on average in 52.37 minutes (SD=20.5). After ten training sessions in the konjac model, the same residents performed Af in an average time of 34.8 minutes (SD=20.5). In this case, the paired mean difference between before and after was a reduction of 17 minutes (95% CI reduction from 25.7 to 11.3 minutes), and this difference was considered significant (p<0.05) (Figure 3).

**Figure 4:**
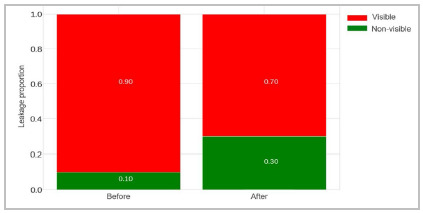
Complementary bar chart with visible leakage ratio before and after training.

As for the presence of leakage, before receiving training it was visible in the anastomoses performed by nine of the 10 residents. After the training sessions, there was a 20% reduction in this figure, though not statistically significant (chi-square=3, p=0.14) (Figure 4).

**Figure 5:**
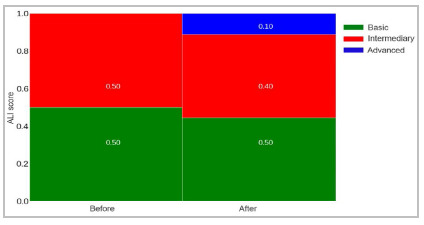
Mosaic chart with the ALI score of residents when performing anastomosis on the placental model before and after performing the konjac training.

Finally, when comparing the ALI, we observed that, before training and after performing A0, five residents had a beginner score and five intermediate. After the experiment, we observed that four residents had a score classified as novice, four as intermediate, and one as advanced. In this case, the move between classes was not significant (Cochran’s t=3, p=0.22) (Figure 5).

## DISCUSSION

Surgical training in general is largely based on a form of learning which consists of the guidance of an apprentice from a senior surgeon, based on cases and situations that appear in clinical practice at random^12^. However, rising hospital costs, medical-legal issues, and concern for patient safety - to name a few - have challenged this training model.

In this context, new methods have been evaluated to achieve the necessary surgical dexterity before their application in the clinical context. Traditionally, live models (mainly mice) have been considered the gold standard. Yasargil himself, the precursor of microsurgical neurosurgery^13^, wrote that to master the technique in microsurgery, a large amount of time in preliminary laboratory practice on animals would be necessary, suggesting a period of daily training for at least six months^1^. Among other advantages, live models allow the surgeon in training to experience the coagulation process in vivo as well as the late test of the anastomoses based on the follow-up of the animal’s health^7^ ^,^ ^14^. However, some challenges, such as the regulation of animal use in research, the ethical evaluation process that is increasingly more careful and detailed, and the high cost of maintaining vivariums are obstacles to the comprehensive use of this model^14^.

With that in mind, following the principle of the 3 “R’s” - replacement, reduction, and refinement - alternative models for animals in experimental research have been sought^15^. In the particular case of microsurgical training of vascular anastomoses, in addition to having the qualities described above, the ideal model should be similar to the real vascular wall^7^. This is a disadvantage of the initial methods frequently used in the initial stages of microsurgery courses, such as latex or silicone tubes^16^. Konjac noodle acquires this property with a properly shaped lumen in its cylindrical structure. The method previously described to perform this task used a 24G (0.7mm) peripheral venous catheter^7^. In this study, however, the attempt to use this caliber was unsuccessful, generating a very narrow lumen, although much easier to perform. A plausible reason is the fact that the noodles used in our study are thicker (~3mm, Figure 2C) than the previously described one (1.5mm). Thus, we prefer to use a 1.1mm (20G) venous catheter, with which an adequate wall: lumen ratio can be created, although it is more difficult to prepare because it is easier to break the noodle structure during the introduction of the catheter, requiring care and performance under microscopy, which in our view constitutes an additional benefit and method for training and familiarization with the manners under the microscope. The noodles are kept in a solution that comes in their packaging, which adds moisture to their wall, giving greater similarity to the appearance found in biological models.

As far as we could verify in the literature, this is the first work designed to: 1) prospectively analyze the learning curve of neurosurgery residents training in the konjac model and 2) evaluate the translation of skills acquired with training in this model in improving the parameters of a termino-terminal anastomosis performed in another more consecrated and already established model (placental artery).

We observed an improvement in the time taken to perform the anastomoses in the placenta after the training sessions in konjac (Figure 3). This trend was similarly detected in other training protocols^17^. When analyzing the complete curve, including the training sessions (not shown, available in supplementary material), there is an increase in Tf compared with the time performed in the last session on the noddle. A study comparing the similarities between konjac and rat artery also observed the times recorded in noodles being faster than those from the animal model^7^. Despite this, the mean Tf was lower than the T0, reflecting a translation, at least in terms of improvement over time, from practice in the konjac model to the placental artery model.

We did not observe a significant difference in ALI score improvement after training in this alternative method. Five residents had their ALI0 considered as novices and five as intermediates. After the ten training sessions, the ALIf of three residents improved, five remained the same, and two worsened. The effect size was probably not large enough to generate statistical significance. However, it is worth noting that the most common error found in the original ALI study was misalignment of the suture line^11^, which was also the most frequent error in our study among A0. This basic error has been corrected, not being seen in the Af.

As mentioned, flow qualities (patency, leakage) are critical for vascular anastomoses. These important characteristics are considered as a limiting step in the transition to the clinical scenario, with some authors proposing a minimum threshold (as high as 80%) of successful results achieved in the in vivo animal model before starting the execution in surgical practice^9^. In inanimate models, these variables are evaluated by different methodologies, such as the use of dyes, among which we can mention colored alcoholic chlorhexidine^7^, colored liquid latex^17^, and more advanced angiography with indocyanine green (ICG)^18^. The use of fluorescein was recently described specifically in vascular anastomoses training^19^. Both ICG and fluorescein are used intraoperatively^20^. Thus, the use of these modalities instead of the more basic ones from the laboratory training phase has the theoretical benefit of familiarizing the future neurosurgeon with such methods. In the case of fluorescein, there is the additional benefit of having a much more affordable cost than the ICG, as well as the necessary apparatus for the fluorescent visualization mode.

In our study, the vast majority of A0 showed gross leakage in the fluorescein test, possibly reflecting the poor quality of these initial anastomoses. The modest improvement verified with this test (only 20% reduction in verified leakage) after the training sessions in konjac may reveal the difficulty in obtaining good quality anastomoses in biological models, even more so in clinical practice. Surgeons who perform cerebrovascular bypasses usually dedicate additional years of training in fellowship programs after completing their medical residency, devoting many hours to laboratory work. For pedagogical reasons, however, we must bear in mind that the objective value intrinsic to the assessment enclosed in the ALI score - which provides corrective feedback for technique errors - cannot be replaced by the patency/leakage testing methodologies of colorimetric infusion methods^17^. We believe that future studies specifically evaluating the correlation between the ALI score and the method for verifying patency/leakage (such as fluorescein) will contribute to improving the quality of vascular anastomoses training.

Finally, although other more widespread methods such as chicken cuts (thigh, wing) and the placenta itself are considered an intermediate alternative that is closer to the animal model from the biological point of view, their use requires services that have a laboratory outside the operating room, for reasons of maintaining a sterile environment. Many training and residency centers only have the existing microscope in the operating room, thus limiting the use of animal sections as training in this environment. The konjac model has already been tested from this point of view, remaining sterile in bacteriological and fungal analysis until close to D14, with the Comitte against noscomial infections allowing its use in the OR^7^. As for the use in simpler household microscopes, the cost benefit is assured, since a package can provide more than 100 anastomoses and food waste is less impactful than that related to the disposal of animal protein after using the chicken wing/thigh.

Some limitations of our study can be pointed out. Regarding the sample, we included Neurosurgery residents from the 1^st^ to the 5th year of training. Despite the relative heterogeneity, all are considered inexperienced as to making anastomoses, since this is not a skill routinely included in the practical training of the specialty.

As already mentioned, the number of participating residents (n=10) may not have been sufficient to statistically demonstrate the improvement trend observed in the qualitative analyzes (fluorescein and ALI leakage). Analogously, the number of training sessions with noodles may also have influenced the qualitative result of the final product in the placenta. At the time of the project’s methodological design, there was no consensus in the literature about the ideal number of anastomoses that should be performed in training before considering the apprentice capable of advancing to the clinical scenario. A recent publication, based on an international consensus on the minimum requirements for courses in microsurgical anastomosis, established 55 anastomoses performed in the laboratory as a criterion^15^. There was, however, no consensus regarding the ideal proportion between practice in non-living versus live models^15^. Thus, at the time of study design, as it was an initial study, 10 sessions were considered appropriate. The increase in the number of training sessions could in theory benefit the final anastomoses. Thus, future studies that recruit a larger number of participants and/or increase the number of training sessions can resolve this issue.

## CONCLUSION

Microsurgical training in the konjac noodle model was associated with improved time to perform an end-to-end microsurgical anastomosis in a human placental model. Other parameters such as a technical error checking (ALI) score and anastomotic leakage did not display significant alterations. New studies with larger samples will be necessary to deepen our understanding of the proposed model. However, konjac ‘s low cost, ease of storage, and tactile feedback similar to other biological models are promising characteristics, which qualify it as an option for training in microsurgery, especially in the initial cycles.
